# Targeted Delivery of siRNA Lipoplexes to Cancer Cells Using Macrophage Transient Horizontal Gene Transfer

**DOI:** 10.1002/advs.201900582

**Published:** 2019-09-04

**Authors:** Elizabeth C. Wayne, Christian Long, Matthew J. Haney, Elena V. Batrakova, Tina M. Leisner, Leslie V. Parise, Alexander V. Kabanov

**Affiliations:** ^1^ Center for Nanotechnology in Drug Delivery and Division of Pharmacoengineering and Molecular Pharmacuetics Eshelman School of Pharmacy University of North Carolina at Chapel Hill Chapel Hill NC 27599 USA; ^2^ Biochemistry and Biophysics University of North Carolina at Chapel Hill Chapel Hill NC 27599 USA; ^3^ Laboratory of Chemical Design of Bionanomaterials Faculty of Chemistry M.V. Lomonosov Moscow State University Moscow 119992 Russia

**Keywords:** cancer, gene delivery, immunotherapy, macrophages, small interfering RNA (siRNA)

## Abstract

Delivery of nucleic acids into solid tumor environments remains a pressing challenge. This study examines the ability of macrophages to horizontally transfer small interfering RNA (siRNA) lipoplexes to cancer cells. Macrophages are a natural candidate for a drug carrier because of their ability to accumulate at high densities into many cancer types, including, breast, prostate, brain, and colon cancer. Here, it is demonstrated that macrophages can horizontally transfer siRNA to cancer cells during in vitro coculture. The amount of transfer can be dosed depending on the amount of siRNA loaded and total number of macrophages delivered. Macrophages loaded with calcium integrin binding protein‐1 (CIB1)‐siRNA result in decreased tumorsphere growth and decreased mRNA expression of CIB1 and KI67 in MDA‐MB‐468 human breast cancer cells. Adoptive transfer of macrophages transfected with CIB1‐siRNA localizes to the orthotopic MDA‐MB‐468 tumor. Furthermore, it is reported that macrophage activation can modulate this transfer process as well as intracellular trafficking protein *Rab27a*. As macrophages are heavily involved in tumor progression, understanding how to use macrophages for drug delivery can substantially benefit the treatment of tumors.

## Graphical abstract

**Macrophage mediated horizontal siRNA transfer** provides an incredible opportunity to deliver therapeutic oligonucleotides (i.e., siRNA, mRNA, microRNA, pDNA) to macrophage‐rich disease environments such as cancer. This work provides a basis for the mechanism of macrophage gene transfer via activation dependent extravesicular vesicle release and thus lays the groundwork for development of nanocomplexes that can control macrophage activation and support gene delivery.

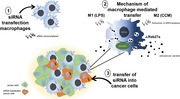

## 1 Introduction

Nanotechnology has been transformative for the efficacy of cancer treatment. Through the precise engineering of nanoparticles, one can increase drug circulation half‐life, synergize combinatorial therapies, and deliver insoluble drugs. However, nanoparticles can have relatively poor tumor distribution, poor endosomal escape. The development of new particles alone may not always overcome the barrier to achieving efficient drug delivery into solid tumors.^1^ This is especially true for nucleic acid‐based therapeutics, where tumor delivery and endosomal escape of oligonucleotides are particularly challenging. The enhanced permeability and retention (EPR) effect, which the promise of cancer nanotechnology has partially relied on, has proved more heterogeneous and unpredictable than anticipated. In contrast to the limitations of nanoparticles, immune cells are an optimal drug delivery vehicle due to their ability to cross‐navigate the blood and lymphatic circulatory system, penetrate the blood–brain barrier, the fibrotic exterior of a tumor, or other barriers, and actively accumulate in diseased regions.^2^, ^3^ Combining the benefits of nanoparticle encapsulation and stability with the intrinsic trafficking, phagocytosis, and secretion activity of immune cells may be the best combination to achieve therapeutic doses.

Targeted delivery of RNA interference (RNAi) therapeutics to the tumor microenvironment has been aggressively pursued but is technically challenging.^4^, ^5^ Small interfering RNA (siRNA) therapeutics are highly effective but are currently limited clinically by poor pharmacokinetics and can benefit from cell carriers. siRNA therapies can target with high precision previously “undruggable” mutations because it can knockdown genes post‐transcription/pre‐translation stage. Further, siRNA therapy avoids the possible genomic mutations associated with DNA therapies.^5^ Because naked siRNA has a short half‐life in circulation, strategies to overcome this limitation involve nanoparticle delivery^6^, ^7^, ^8^ or structural modification of the siRNA itself.^9^ However, these limitations of siRNA pharmacokinetics do not impede their direct delivery to the eye, lung, and liver.^7^, ^10^ One recent example is Patisiran (Alnylam), an RNAi therapeutic that targets a protein produced in the liver, has completed Phase 3 trials and is the first RNAi therapeutic to receive FDA approval.^11^ Still, many cancer‐related RNAi therapies are limited by effective transport into solid tumors and endosomal escape once inside the cell.

Cationic siRNA lipoplexes are beneficial packaging systems for nonviral siRNA delivery.^12^, ^13^, ^14^ Cationic lipids form complexes with negatively charged siRNA via electrostatic interaction.^15^, ^16^ Optimization of the complex formation parameters (such as lipid composition, ratio to siRNA, mixing temperature, etc.) has led to efficient delivery into the cell and subsequent endosomal escape in vitro.^17^, ^18^ However, due to cationic lipid‐induced toxicity, inflammation, and poor distribution profiles, in vivo success of lipoplexes has been limited.

Using immune cells as drug delivery vehicles has been well published.^19^, ^20^, ^21^ Immune cells have been used as broad functional carriers, where the surface is coated with therapeutic nanoparticles. This method has been employed to target white blood cells within the bloodstream,^22^ T lymphocytes,^23^ natural killer cells^24^ among others. Red blood cells have been used to carry drugs^25^ and have been very useful in delivering drugs to blood clots and injury sites because of their long lifespan and large quantity. The methods previously described involve incorporation of nanoparticles to the surface of the immune cells and rely passively on the migratory properties of the immune cells. However, another type of packaging which requires a functional interaction between the nanoparticle cargo and the immune cells. This describes cells such as macrophages that can internalize their delivery cargo and release at the disease site. Macrophages in other contexts have been shown to transfer oligonucleotides to surrounding cells.^26^, ^27^ Our previous work has shown that macrophages can horizontally transfer proteins and DNA into neurons,^28^ muscle cells,^29^ and even immune cells in distal organs.^30^

There are two central reasons why macrophages are ideal carriers for delivery into solid tumors. First, macrophages penetrate and accumulate in tumors at significant numbers throughout tumor progression. This accumulation can account for 30–50% of the mass of a solid tumor. Many immunotherapies centralize T cells; however, there are several reasons why this strategy is limiting. It has been well documented that T‐cells either do not localize or cannot survive the tumor suppressive environment.^31^, ^32^ While T‐cell therapies have shown success in blood cancers such as leukemia and non‐Hodgkin's lymphoma, these therapies leave out 80% of the patient population who present with solid tumors. Even within blood cancers, only subset of those patients have sufficient numbers of T cells that can be harvested.^33^ Since macrophages participate in antigen presentation, targeting macrophages may also have the benefit of priming the solid tumor environment for T‐cell infiltration. Second, macrophages phagocytose and secrete oligonucleotides and proteins into the surrounding environment. Intercellular communication can modulate the micro or local environment via secretion of cytokines and nucleic acids. However, it is less well appreciated that macrophages can transfer exogenously administered proteins and oligonucleotides. Considered together, macrophages pose an advantageous and efficient delivery carrier for the tumor microenvironment.

In this study we demonstrate that macrophages horizontally transfer siRNA to surrounding cells in both 2D and 3D tumorsphere coculture models. Macrophages that were adoptively transferred into mice were also able to penetrate the tumor micronenvironment and transmit pharmacologically active siRNA to cancer cells in an orthotopic MDA‐MB‐468 breast cancer model. This work propels the general use of macrophages for therapeutic delivery and provides a new model for drug delivery into solid tumor masses that have been notoriously difficult for nanoparticle and other immunotherapies to penetrate.

## 2 Results and Discussion

### 2.1 Characterization of siRNA Lipoplex Loading into Macrophages

**Figure 1:**
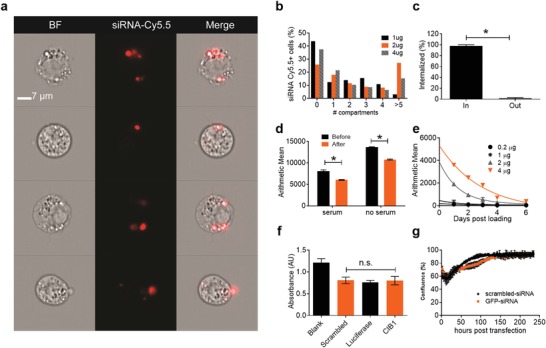
Characterization of siRNA loading into IC 21 macrophages. a) Imaging Flow Cytometry (Imagestream) image of IC21 macrophages loaded with scrambled siRNA‐Cy5.5. Objective 60×; scale bar 7 µm. b) Histogram of the percent of cells with different numbers of compartments (vesicles) containing scrambled siRNA‐Cy5.5 24 h after transfection. c) Imagestream analysis of degree of internalization of scrambled siRNA‐Cy5.5 nanoformulation immediately after macrophage transfection and heparin sulfate wash. d) FACS analysis of mean intensity of macrophages transfected with scrambled siRNA‐Cy5.5 before and after wash with 1 mg mL^−1^ heparin sulfate. e) 1 × 10^6^ IC21 macrophages were plated in a 6 well plate transfected with varying concentrations of scrambled siRNA‐Cy5.5 + 0.2 µg (black); 1 µg (asterisk); 2 µg (grey, triangle), 4 µg (orange, upside‐down triangle). Plotted lines represent exponential decay fitted curves used to calculate siRNA half‐life. FACS analysis of siRNA‐Cy5.5 remaining in macrophages up to 6 days after loading. f) CCK‐8 Cytotoxicity assay 10 × 10^4^ IC21 macrophages were plated into a 96 well plate and loaded with either scrambled siRNA‐Cy5.5, Luc siRNA, or CIB1‐siRNA. g) 10 × 10^4^ IC21 macrophages were plated into a 96 well plate and loaded with either scrambled siRNA‐Cy5.5 or GFP siRNA. Time‐lapse images were taken every 2 h for 7 days and the proliferation (percent of area covered by cells) was analyzed. Statistical analysis done using Prism software by one‐way ANOVA followed by post hoc Tukey test. c,d) Unpaired *t*‐test for two group comparisons. **p < 0.005; *n* = 3 for all groups

To demonstrate proof of transient horizontal transfer, commercially available cationic transfection lipoplexes (GeneSilencer; Genlantis) were used to load scrambled, nonhomologous siRNA labeled with Cy5.5 (Dharmacon) into the macrophages.^34^, ^35^, ^36^ Negative Stain TEM imaging and nanoparticle tracking analysis (NTA) revealed condensed siRNA lipoplexes with average sizes of 177.2 nm ± 6.6 nm (Figure S1, Supporting Information). Imaging flow cytometry (Imagestream, Millipore) analysis confirmed that the siRNA lipoplexes were found in intracellular vesicles (**Figure** **1**a). There was a heterogeneous distribution in the uptake amount of siRNA lipoplexes (Figure 1b). The siRNA lipoplexes were fully internalized with less at 0.01% of siRNA lipoplex signal colocalizing with the cellular membrane (Figure 1c). Following transfection, macrophages were rinsed with 1 mg mL^−1^ heparin sulfate to dissolve noninternalized lipoplexes. Measurements of macrophages before and after heparin sulfate wash reveal significant differences in the mean intensity (Figure 1d). The amount of Cy5.5‐labeled‐siRNA found within the cell was proportional to the initial loading concentrations (Figure 1e). The mean intensity data was fit to an exponential decay curve to calculate the half‐life of siRNA within the macrophage populations. The half‐life of the siRNA in the macrophages increased with higher loading concentrations from 1.3 to 2.1 days for 0.2 to 4 µg respectively. This value encapsulates population levels dynamics of siRNA transfer, degradation, and cell division—how these factors individually affect the half‐life of siRNA within macrophages should be further studied.

Cytotoxicity due to siRNA loading was measured using the CCK‐8 assay. The absorbance was higher in the nonloaded macrophages but similar across all three siRNA constructs tested (Figure 1f). To observe the effect of siRNA loading on the proliferation, macrophage cell growth was measured over 7 days and found no significant differences in growth rate between Luciferase siRNA (Luc siRNA) and scrambled siRNA loaded macrophages (Figure 1g). Thus, the macrophage proliferation and survival behavior were unaffected by the loading with siRNA lipoplexes. In addition, we characterized siRNA loading into RAW 264.7 cells and found similar trends with regards to dosing and cytotoxicity (Figure S2, Supporting Information).

### 2.2 Luc siRNA Horizontal Transfer from Macrophages Results in Knockdown of Luciferase in the Cancer Cells

**Figure 2:**
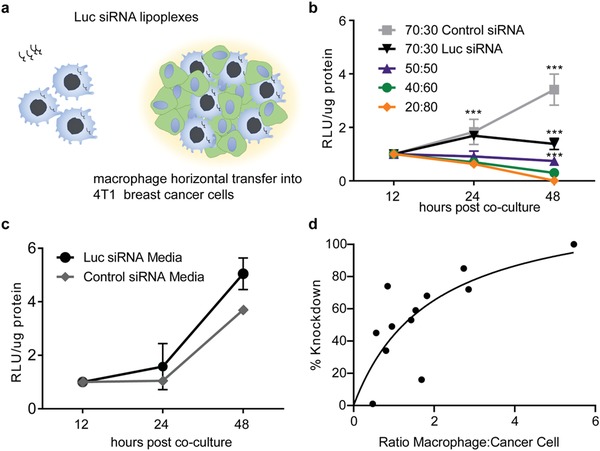
Macrophage horizontal transfer of Luc siRNA into cancer cells. a) Schematic of siRNA transfer coculture experiment. Balb/c RAW 264.7 macrophages transfected with 4 µg of Luc siRNA or 4 µg of scrambled siRNA (control siRNA). Twenty‐four hours after transfection, macrophages are rinsed and cocultured with Balb/c 4T1 breast cancer cells stably expressing Renilla‐Luciferase (4T1‐RLuc) at varying compositions. b) The luciferase activity was measured at different timepoints after coculture incubation and was expressed in relative light units (RLU) normalized by total protein content. A composition of 70:30 corresponds to a total composition of 70% cancer cells and 30% macrophages. c) Media removed from RAW 264.7 macrophages prior to heparin sulfate wash was collected and incubated with 4T1‐RLuc cancer cells. RLU µg^−1^ protein values are calculated and normalized to the 12 h timepoint. d) Percentage of luciferase knockdown calculated for each condition at different time points and graphed as a function of the ratio of macrophages to cancer cells at that timepoint. The number of cells at different time points were estimated using macrophage and cancer cell doubling times. ANOVA statistical analysis performed with Tukey posttest. ***p < 0.0001; *n* = 3 for each timepoint

The transfer of Luc siRNA from macrophages to the cancer cells was examined by measuring the reduction of luciferase bioluminescence activity in the cancer cells following coculture with Balb/c RAW 264.7 macrophages transfected with Luc siRNA (**Figure** **2**a). As the number of macrophages increased, the knockdown also increased (Figure 2b), demonstrating evidence of siRNA transfer that was dependent on the ratio of macrophages and the duration of coculture. As a negative control, macrophages were also transfected with an equal amount of scrambled, nonhomologous siRNA (control‐siRNA). There was no reduction in luciferase activity when cancer cells were cocultured with control‐siRNA which suggests cancer cell death is due to coculture is not likely occurring. Furthermore, no difference in luciferase activity (Figure 2c) was noted in cancer cells incubated in media harvested from macrophages 24 h after transfection with either control siRNA or Luc siRNA. Interestingly, under conditions of this experiment, the maximal knockdown was observed when macrophage to cancer cell ratio was higher than 50% (Figure 2d). Since tumor associated macrophages make up to 50% of tumor mass,^37^, ^38^ this suggests a real potential for macrophages as delivery carriers in cancer treatments.

Macrophage horizontal transfer of siRNA was also tested using other macrophage cell lines. Knockdown of luciferase expression in 3LL Lewis Lung carcinoma cells was replicated in human THP‐1 macrophages (Figure S3, Supporting Information). THP‐1 did not monocytes show effective transfer, and this may be due to their poor siRNA transfection (Figure S4, Supporting Information). Optimization of culture conditions and siRNA transfection parameters will be needed to fully test monocyte gene transfer. Overall, these results using several macrophage and cancer cell models support the finding that the siRNA is being transferred from the macrophage to the cancer cells and suppresses gene expression.

### 2.3 siRNA Horizontal Transfer Dynamics

**Figure 3:**
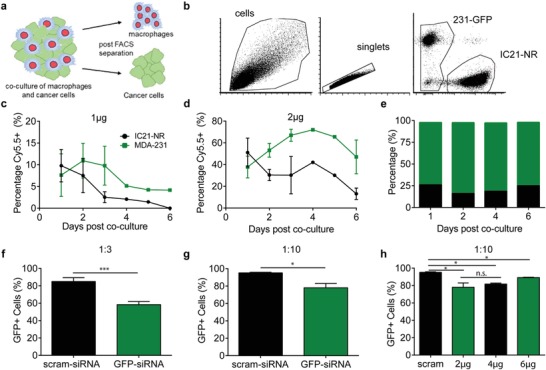
Kinetics of siRNA transfer between macrophages and cancer cells. a) Schematic of the study. b) Flow cytometry separation analysis of macrophages and cancer cells. Plots show (left to right) all measured live cells, single cells, and distinct distribution of MDA‐MB‐231 GFP cancer cells and IC21‐NR macrophages. IC21‐NR macrophages (1.0 × 10^6^) are transfected with either c) 1 µg or d) 2 µg of scrambled siRNA labeled with Cy5.5. Immediately following transfection, the macrophages are rinsed and mixed with MDA‐MB‐231‐GFP cells in rounded, ultralow attachment 96 wells at a 50:50 cell ratio. Then, the seeded 96 well plates were centrifuged at 1500 × *g* for 15 min to initiate tumorsphere formation before placing into a 37 °C incubator. At different timepoints, the tumorspheres were collected, disaggregated, and analyzed via flow cytometry. c,d) Percentage of macrophage (black) and cancer cell (green) populations containing Cy5.5‐labeled scrambled siRNA. e) The average percentage of measured cells that were macrophages (black) or cancer cells (green) in the coculture at different timepoints. f,g) IC21‐NR macrophages (1.0 × 10^6^) are transfected 2 µg of GFP‐siRNA and cocultured with MDA‐MB‐231‐GFP cells at a ratio of f) 1:3 or g) 1:10. h) IC21‐NR macrophages (1.0 × 10^6^) are transfected with 2, 4, or 6 µg of GFP‐siRNA and cocultured with MDA‐231‐GFP cells. f–h) The percentage of GFP+ expressing cells were analyzed 48 h after coculture using flow cytometry. Statistical analysis is done by unpaired *t*‐test using Prism software: **p < 0.005; ***p < 0.001; *n* = 3 for each measurement

The Luc siRNA assay provided a sensitive tool for demonstration of siRNA transfer from a macrophage donor to a cancer cell recipient; however, it was difficult to understand the kinetics of this transfer without quantifying cell population numbers and the amount of siRNA within each group. Therefore, a fluorescent coculture model was developed (**Figure** **3**a). IC21 macrophages were stably transduced with a nuclear localizing red fluorescent protein, NucLight Red (Essen Bioscience) lentivirus (IC21‐NR) and MDA‐MB‐231 cancer cells were stably transduced to express GFP (MDA‐MB‐231‐GFP). The two cell populations remain distinct during coculture evidenced by flow cytometry analysis (Figure 3b).

We further characterized our coculture model by investigating the effect of coculture on cell viability on MDA‐MB‐231 cells. Using time‐lapse fluorescent microscopy, we cocultured the human cells with either human THP‐1 macrophages, C57Bl6 IC‐21 macrophages, or Balb/c RAW 264.7 macrophages. As a control MDA‐MB‐231 GFP cells incubated alone. Viability was assessed by the increase in GFP signal over time. To determine whether cytokine signaling from macrophages affected the cancer cells growth, MDA‐MB‐231 GFP cells were incubated with media from the macrophage cells lines. We found that while the MDA‐MB‐231 GFP cells grew best in the control group, there was no significant difference in proliferation among the macrophage conditioned media treated cancer cells (Figure S5a, Supporting Information).

MDA‐MB‐231 GFP cells were also cocultured with human with macrophages at a 1:2 ratio comparable to experiments within the paper (Figure S5b, Supporting Information). There was a statistically significant difference in proliferation in the coculture experiment between the monoculture control and the macrophages cocultures. However, there was no significant difference between human or mouse media or coculture groups. The difference in proliferation between the cancer cell monoculture control and cocultured with macrophages is likely a result of limited surface area upon which to grow and nutrient resources—the cocultures had more cells (Figure S5c, Supporting Information). We conclude that there is an effect on cancer cell growth due resource competition as a result of coculturing. Importantly, there are no distinguishable effect on viability of culturing human cancer cells with mouse macrophages in comparison to culturing with human macrophages.

IC21‐NR macrophages were transfected with two different concentrations of a scrambled siRNA tagged with a Cy5.5 fluorescent dye (scrambled siRNA‐Cy5.5). Immediately after transfection, macrophages were cocultured with MDA‐MB‐231‐GFP cells in a tumorsphere. At various intervals between 1 and 6 days after tumorsphere initiation, tumorspheres were disaggregated into a single cell suspension and analyzed using flow cytometry to determine the macrophage and cancer cell fractions containing scrambled siRNA‐Cy5.5.

For both macrophage‐loading conditions, the percentage of cancer cells containing siRNA surpassed that of macrophages containing siRNA at around 36 h of coculture. As expected, the macrophages loaded with a higher amount of siRNA (2 µg) showed a higher overall percentage of siRNA positive cells for both the donor and recipient cells. In addition, the percentage of cancer cells containing siRNA peaked at 2 days for the 1 µg loading condition (Figure 3c) and 4 days for 2 µg loading condition (Figure 3d). The overall population of macrophages and cancer cells measured at any given time point remained relatively constant. IC21‐NR macrophages composed 60–80% of the cells while MDA‐MB‐231‐GFP cancer cells composed 20–40% of cells measured (Figure 3e). These data suggest that as the initial macrophage loading increased, the transfer of siRNA from macrophages to the cancer cells and the retention of the siRNA in both cell types also increased.

The knockdown of GFP expression via horizontal transfer of GFP‐siRNA was investigated by loading IC21‐NR macrophages with 2 µg GFP‐siRNA (Thermofisher) and measuring GFP expression in the MDA‐231‐GFP populations 48 h post coculture. Macrophages were cocultured with cancer cells in either a 1:3 (Figure 3f) or 1:10 (Figure 3g) seeding ratio. There was a 31% decrease in GFP+ positive cells in the IC21(GFP‐siRNA) coculture compared negative control coculture (Figure 3f). However, at the lower ratio (1:10) there was only an 18% decrease in GFP+ positive cells (Figure 3g). Interestingly, the macrophage:cancer cell ratio and the percentage knockdown fits within the relationship proposed in (Figure 2d) even though the macrophage cell line and target cancer cells are different.

As a point of comparison, MDA‐MB‐231 cancer cells were directly transfected with GFP‐siRNA using geneSilencer lipoplexes (Figure S6, Supporting Information). The percentage of GFP+ cells decreased by 72% using similar loaded conditions to the macrophage transfer experiment (2 µg GFP‐siRNA, measured 48 h post transfection). While direct transfection was more effective, their capacity for in vivo application is limited due to toxicity. The ability of macrophages to hone to regions of inflammation, carry and transfer function siRNA is a notable combination.

To test the role of initial loading, we kept the coculture ratio at 1:10 but varied the initial amount from 2 to 6 µg of GFP‐siRNA (Figure 3h). While there were decreases in the percentage of GFP+ cells (13% and 6% for 4 and 6 µg, respectively), they did not perform significantly better than 2 µg. This may be due to the saturation of the macrophage loading capacity which plateaus at 2 µg using the geneSilencer transfection system (Figure S7, Supporting Information). In the future we might specifically consider technologies that can increase loading in macrophages such as using mannose‐receptor targeted polyplexes.

### 2.4 Macrophages Loaded with CIB1‐siRNA Inhibit Growth of MDA‐MB‐468 Cells

**Figure 4:**
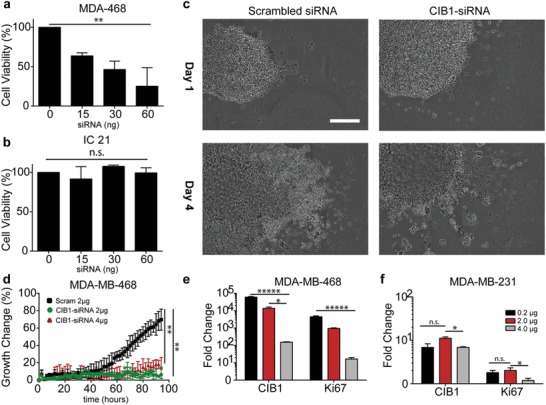
Macrophages loaded with CIB1‐siRNA results in decreased growth of breast cancer cells. a) MDA‐MB‐468 breast cancer cells. b) IC21 macrophages cells were transfected with 0–60 µg CIB1‐siRNA. Forty‐eight hours after transfection cell cytotoxicity was measured using CCK‐8 assay. IC21 macrophages were transfected with either 2 or 4 µg CIB1‐siRNA or 2 µg scram‐siRNA and cocultured at a ratio of 1:2 IC21 macrophage:MDA‐MB‐468 cells in a tumorsphere formation. c) Time‐lapse brightfield images of tumorspheres in coculture containing MDA‐MB‐468 cancer cells and IC21 macrophages loaded with either CIB1‐siRNA or scrambled siRNA at Day 1 (top) and Day 4 (bottom). d) The percentage of tumorspheres growth change was quantified. e,f) IC21 macrophages were transfected with either 0.2, 2, or 4 µg CIB1‐siRNA cocultured a ratio of 1:2 with e) MDA‐MB‐468 or f) MDA‐MD‐231 cells in a tumorsphere formation. e,f) Changes in mRNA expression of CIB1 and Ki67 after 4 days of coculture. One‐way ANOVA analysis was performed with Tukey post hoc test: **p < 0.01; ***p < 0.001; *n* = 3 for all samples

Next, we examined whether the transfer of siRNA from macrophage to cancer cells could result in a therapeutic anticancer effect. Towards this goal, we delivered siRNA to knockdown calcium integrin binding protein‐1 (CIB1) that is known to promote survival and proliferation in triple negative breast cancer (TNBC) cells via regulation of AKT and EKT activation.^39^, ^40^ Consistent with previous reports, the human TNBC MDA‐MB‐468 cells displayed a dose‐dependent decrease in the cell survival after transfection with CIB1‐siRNA (**Figure** **4**a). In contrast, mouse IC21 macrophages were insensitive to CIB1‐siRNA (Figure 4b). Moreover, quantitative real‐time polymerase chain reaction (qPCR) analysis of the macrophages did not show a measurable amount of CIB1 RNA, which made them perfectly suitable as a vehicle for the delivery of CIB1‐siRNA to the TNBC cells. IC21 macrophages were transfected with CIB1‐siRNA and then cocultured MDA‐MB‐468 cells at a 1:2 ratio (one macrophage for every 2 cancer cells) in a tumorsphere as described above to recapitulate the 3D contact of macrophages and cancer cells as seen in vivo. The growth of tumorspheres was analyzed by live cell imaging and expressed as percentage of growth change (Figure 4c). There was a significant reduction in the tumorspheres growth kinetics for the groups containing macrophages transfected with CIB1‐siRNA and those transfected with control siRNA. During the 4 days of coculture the control tumorspheres increased up to 70% in confluency whereas the CIB1‐siRNA treated tumorspheres experienced only a 20% or 30% change in growth (Figure 4d). There was no significant difference between the two initial loading concentrations of CIB1‐siRNA (2 or 4 µg) on the effect on tumorsphere growth, albeit the higher CIB1‐siRNA concentration trended toward increased growth inhibition. Overall however, both CIB1‐siRNA groups show smaller and more compact tumorspheres than control siRNA groups 4 days post coincubation.

In a separate study, tumorspheres were analyzed for changes in mRNA expression of CIB1 and KI67, a marker of human cell proliferation. Coculture of MDA‐MB‐468 breast cancer cells with IC21 macrophages transfected with varying amounts of CIB1‐siRNA resulted in significant decreases in CIB1 and KI67 mRNA expression (Figure 4e). In contrast, MDA‐MB‐231 cells were not sensitive to CIB1 signaling.^39^, ^40^ Coculture of MDA‐MB‐231 cells with IC21 macrophages transfected with CIB1‐siRNA did not significantly reduce expression of CIB1 or KI67 (Figure 4f). To test the effect of gene transfer on nornal cells, MCF10a (human epithelial breast cells) were also cocultured with IC21 macrophages transfected with 2 µg CIB1‐siRNA. No significant differences in CIB1 or KI67 mRNA expression were found (Figure S8, Supporting Information).

### 2.5 Macrophage Activation Facilitates Horizontal Transfer of siRNA

**Figure 5:**
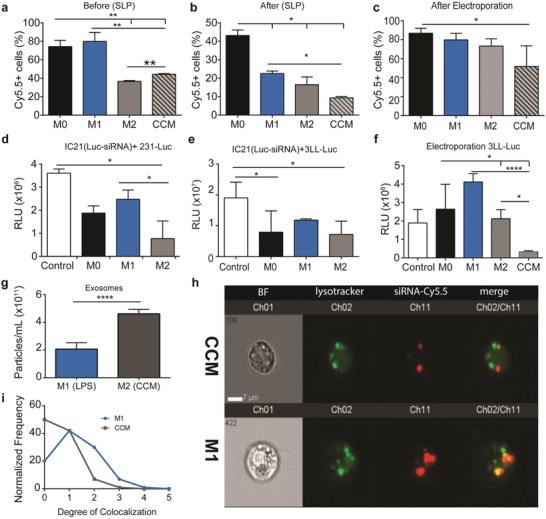
Macrophage activation facilitates horizontal transfer of siRNA. Percentage of IC21 macrophage containing scrambled siRNA Cy5.5 when conditioned with activation media a) 24 h before, or b) 24 h after siRNA lipoplex transfection (SLP), or c) 24 h after transfection using electroporation (EP). Percentages of scrambled siRNA Cy5.5 remaining 24 h a) post transfection or b,c) post conditioning with activation media are presented. Luciferase expression in d) MDA‐231‐Luc or e,f) 3LL‐Luc cells after coculture of these cells for 48 h with activated macrophages carrying Luc siRNA. Macrophages were first activated for 24 h, then transfected with 2 µg Luc siRNA using either d,e) siRNA lipoplex or f) electroporation, and immediately after transfection cocultured with Luciferase‐expressing cancer cells for 48 hours. Luciferase activity was expressed in relative light units (RLU). d–f) Control represents naïve unconditioned macrophages transfected with scrambled siRNA and cocultured with cancer cells. g) Quantification of exosomes collected over 24 hours from M1 (LPS) and M2 (CCM) activated macrophages. h) Macrophages transfected using scrambled siRNA Cy5.5 lipoplex were conditioned with activation media for 24 h and stained with Lysotracker Green. Imagestream colocalization analysis of siRNA and Lysotracker Green between M1 (LPS) and M2 (CCM) activated macrophages. i) Quantification of degree of colocalization histogram. In all panels one‐way ANOVA analysis was performed with Tukey post hoc test: **p < 0.01, ***p < 0.001; *n* = 3 for each group

Because macrophages change their functionality based on local external stimuli, it was important to investigate whether activation informs macrophage horizontal gene transfer. Macrophages were activated into three phenotypes, proinflammatory (M1; 1ug LPS), anti‐inflammatory (M2; 20 ng mL^−1^ IL‐4), or tumor associated macrophages (TAM; cancer conditioned media (CCM)). Naïve, unconditioned macrophages (M0) were used as a control for all experiments. The activation phenotype (**Table** **1**) was characterized by qPCR measurement of mRNA expression of relevant genes (Figure S9, Supporting Information). Macrophages activated 24 h prior to transfection with scrambled siRNA Cy5.5 lipoplexes were found to have differential uptake activity (**Figure** **5**a). M0 and M1 activated macrophages contained comparable percentages of siRNA Cy5.5+ cells, however M1 macrophages had a higher mean intensity indicating that M1 macrophages endocytosed a larger amount of siRNA lipoplexes (Figure S6, Supporting Information). In contrast, both M2 and CCM activated macrophages endocytosed fewer siRNA lipoplexes than M0 and M1.

**Table 1:** Primers used in qPCR experiments. Chart of primers used in qPCR reactions (M denotes a mouse specific gene; H denotes a human specific gene; F forward; R reverse)

18s F	TGTGCCGCTAGAGGTGAAATT
18 R	TGGCAAATGCTTTCGCTTT
M Arg1 F	CTCCAAGCCAAAGTCCTTAGAG
M Arg1R	GGAGCTGTCATTAGGGACATCA
M Mrc2 F	TCTCCCGGAACCGACTCTTC
M Mrc2 R	AACTGGTCCCCTAGTGTACGA
M Nos2 F	ACATCGACCCGTCCACAGTAT
M Nos2 R	CAGAGGGGTAGGCTTGTCTC
M CD163 F	GGTGGACACAGAATGGTTCTTC
M CD163 R	CCAGGAGCGTTAGTGACAGC
M TNFA F	CGTCTCGCAACCTACAAGCA
M TNFA R	GGTATCCGACTCTACCCTTGG
H CIB1 F	ACATCAAGTCCCATTATGCCTTC
H CIB1 R	GACGCACTAAGCCGTGTGT
H Ki67 F	GGGCCAATCCTGTCGCTTAAT
H KI 67 R	GTTATGCGCTTGCGAACCT

The effect of macrophage activation on intracellular trafficking of siRNA lipoplexes by transfecting M0 macrophages with siRNA lipoplexes and exposing to activation conditions for 24 h before analysis. The M1 macrophages contained 56% less siRNA lipoplexes in comparison to naïve, M0 macrophages (Figure 5b). Interestingly, M2 activated macrophages contained 67% less while CCM macrophages contain 82% less (Figure 5b). To elucidate the fate of the siRNA lipoplexes, lysosomal activity was measured. Colocalization studies using imaging flow cytometry revealed that siRNA lipoplexes M1 activated macrophages had higher colocalization with Lysotracker Green (Invitrogen) (Figure 5i) than CCM activated macrophages. Quantified over 10 000 cells for each condition (Figure 5h), M1 macrophages had on average a colocalization score of 2.5 in comparison to CCM (≈1.2). Moreover, CCM activated macrophages secreted higher amounts of exosomes in comparison to M1 activated macrophages (Figure 5g). Finally, macrophages activated into an M2 phenotype and loaded with siRNA lipoplexes exhibited a higher degree of siRNA transfer than M1 macrophages to both MDA‐MB‐231‐Luc human breast cancer cells (Figure 5d) and 3LL‐Luc murine Lewis lung carcinoma cells (Figure 5e).

To test the effect of the lipoplex in gene transfer, macrophages were transfected via electroporation. IC21 macrophages were electroporated with 2 µg of scrambled siRNA Cy5.5 and exposed to activating media for 24 h before analysis with flow cytometry (Figure 5c). Results show a similar trend to lipoplex‐transfected macrophages in (Figure 5b) although there was a higher percentage of cells containing siRNA Cy5.5 in all groups. In addition, IC21 macrophages electroporated with 2 µg of Luc siRNA and cocultured with 3LL‐Luc cancer cells demonstrated similar trends in knockdown of luciferase activity (Figure 5f). This suggests that the lipoplex may affect intracellular trafficking but that macrophage horizontal gene transfer is also linked to the activation phenotype of the macrophage.

### 2.6 Horizontal Transfer Facilitated via Rab 27a Recycling Pathway

**Figure 6:**
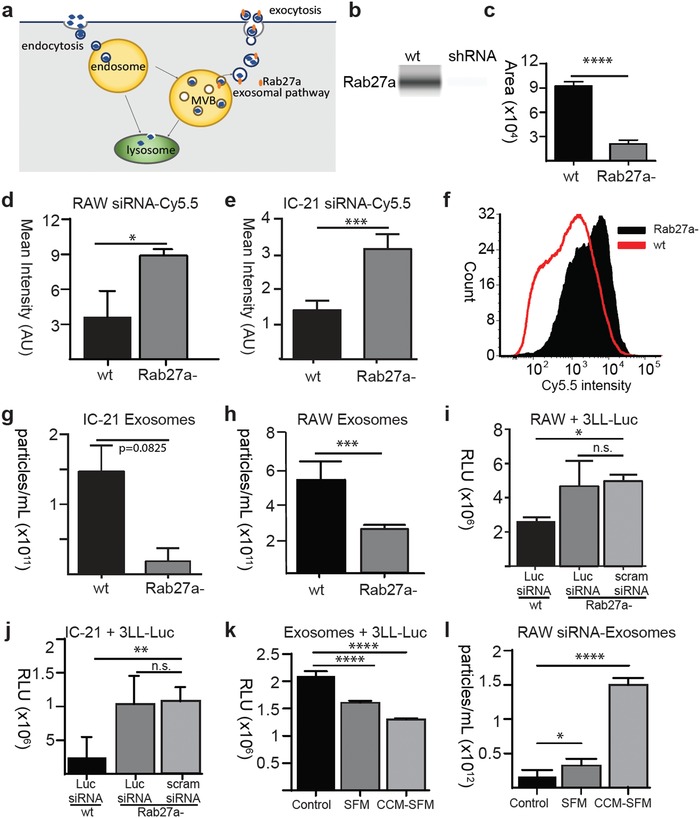
Horizontal transfer facilitated via Rab27a recycling pathway. a) Schematic of exosomal secretory pathway. b) Western blot and c) quantification of *Rab27a*‐ protein expression in IC21 wildtype (*wt*) and Rab27a‐shRNA (*Rab27a‐*) stably transduced knockdown cell line. Arithmetic mean intensity of scrambled siRNA Cy5.5 remaining in d) RAW 264.7 *wt* or *Rab27a‐* and e) IC21 *wt* or IC21 *Rab27a‐* macrophages 24 h after lipoplex transfection. f) Histogram of distribution of siRNA Cy5.5 intensity between IC21 *wt* (red) or IC21 *Rab27a‐* (black) macrophages. Quantification of exosomes collected over 24 h from g) IC21 *wt* or IC21 *Rab27a‐* macrophages. h) RAW 264.7 *wt* or *Rab27a‐* macrophages. 3LL‐Luc cancer cells were cocultured with either i) RAW 264.7 *wt* or *Rab27a‐* or j) IC21 *wt* or IC21 *Rab27a‐* macrophages that were transfected with Luc siRNA or scrambled siRNA. Bioluminescence activity (RLU) was measured 48 h after coculture. k) RAW 264.7 macrophages were transfected with Luc siRNA and incubated nonconditioned serum free media (SFM) or cancer conditioned serum free media (CCM‐SFM) for 24 h prior to exosome isolation. Exosomes were collected and incubated with 3LL‐Luc cells. Bioluminescence activity (RLU) was measured 48 h after coculture. l) Quantification of exosomes harvested after macrophages were transfected with Luc siRNA. Statistical analysis done using Prism software by Unpaired *t*‐tests. *p < 0.05, **p < 0.01, ***p < 0.001, ****p < 0.001, n.s. = nonsignificant; *n* = 3 for each sample group

Because of the finding of enhanced exosomal secretion in M2‐activated macrophages, exosomal trafficking pathways were investigated. *Rab27a* is a trafficking protein that regulates the intracellular exosome secretion pathway (**Figure** **6**a).^41^, ^42^ We stably transduced IC21 and RAW 264.7 macrophages to express *Rab27a*‐shRNA. Western blot analysis confirms knockdown of *Rab27a* protein expression in the knockout cell line (Figure 6b,c). IC21 and RAW 264.7 cell lines were transfected with 2 µg of scrambled siRNA Cy5.5 lipoplexes and measured for the mean intensity 24 h later. In both cell types, *Rab27a‐* macrophages retained more siRNA than *wt* macrophages (Figure 6d,e) and exhibited a narrower spectral distribution of the mean intensity macrophage (Figure 6f). Moreover, there were fewer exosomes isolated over a 24 h period from RAW 264.7 (Figure 6h) and IC 21 *Rab27a‐* (Figure 6g) macrophages in comparison to IC21 *wt*. *Rab27a‐* macrophages transfected with 2 µg of Luc siRNA and cocultured with 3LL‐Luc (Figure 6i,j). Neither cell type cancer cells had a significant knockdown in luciferase activity after 48 h.

In Figure 5, it was demonstrated that macrophage activation enhanced siRNA transfer. A possible mechanism is through enhanced exosomal secretion under M2, anti‐inflammatory macrophage activation, particularly when exposed to cancer conditioned media. To test this hypothesis, RAW 264.7 *wt* macrophages were transfected with Luc siRNA and rinsed with heparin sulfate to remove remaining extracellular lipoplexes. Macrophages were then incubated in either nonconditioned serum free media (SFM) or cancer conditioned serum free media (SFM) for 24 h. As a control, nontransfected macrophages were incubated in nonconditioned serum free media. Exosomes were isolated from the media via polyethylene glycol (PEG) precipitation, collected in 100 µL of DMEM. Twenty microliters of exosomal solution was incubated with 3LL‐Luc cells. After incubation, we found that there was decreased luciferase activity in the SFM and CCM‐SFM exosome treated groups in comparison to the control group (Figure 6k). Moreover, the CCM‐SFM group had 23% lower luciferase activity than the SFM group, suggesting higher efficacy. Quantification of the exosomes reveal that CCM‐SFM had a higher concentration of exosomes than the SFM or control groups (Figure 6l). Taken altogether, this strongly suggests that exosomal secretion via M2 activation is involved with gene transfer.

### 2.7 CIB1‐siRNA Loaded IC21 Macrophages Infiltrate and Transfer siRNA into MDA‐MB‐468 Tumors

Finally, macrophage horizontal transfer of siRNA was tested in an in vivo mammary tumor model. IC21 macrophages were used in the in vivo model because the kinetics of siRNA transfer has already been well characterized in the previous in vitro models. There is no evidence that the macrophages themselves cause cytotoxicity to cells in our coculture experiments. In addition, the CIB1‐siRNA construct has already been validated and characterized to decrease MDA‐MB‐468 tumor growth in nude animal models in previous literature.^39^, ^40^ Moreover, previous preclinical studies have delivered mouse macrophages in conjunction with nude mouse models inoculated with human cancer cells.^43^, ^44^ We used of the macrophage and human cancer cell lines used in the in vitro tumorsphere assays in (Figure 4) in the paper.

**Figure 7:**
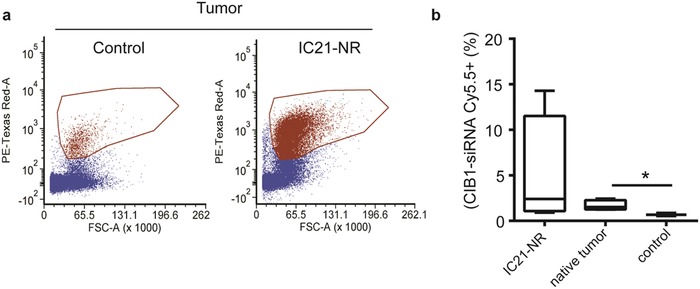
Adoptively transferred IC21 macrophage infiltrate MDA‐MB‐468 tumor and horizontally transfer CIB1‐siRNA. Groups of six female nude mice were inoculated with MDA‐MB‐468 human breast cancer cells. Mice were intravenously injected with 1.0 × 10^6^ IC21‐NR macrophages transfected with 1 µg CIB1‐siRNA‐Cy5.5, five weeks after tumor inoculation. Twenty‐four hours later, tumors were resected and digested to quantify percentage of cell populations within the tumor. a) FACS analysis of separation of adoptively transferred IC21‐NR macrophages (right) and cells found within the tumor (left). b) Box plot presents the percentages of adoptively transferred IC21‐NR cells from the tumor and the percentage of all remaining cells found within the tumor that are positive for CIB1‐siRNA Cy5.5. The control represents the tumor cells isolated from control groups that were not adoptively transferred with IC21‐NR macrophages. The median value of the boxplot is displayed as a line while the top and bottom of the box mark the limits of ±25% of the variable population. Statistical analysis by unpaired *t*‐test using Prism software: *p < 0.05; *n* = 5 for each group

MDA‐MB‐468 cells were orthotopically implanted into nude mice and monitored weekly. At 5 weeks, mice were injected with naïve, non‐activated IC21 macrophages labeled with Nuclight Red (IC21‐NR), a nuclear localizing red protein that allows distinction between native and adoptively transferred macrophages. Mice were sacrificed 24 h after injection and the primary tumors were resected and weighed before digestion. Flow cytometry analysis of digested tumor cells positively confirms an infiltration of IC21‐NR macrophage into the tumor (**Figure** **7**a). Further analysis shows a correlation between the size of the tumor and the percentage of IC21‐NR macrophage infiltration. Similar to the experimental scheme in (Figure 2), the percentage of CIB1‐siRNA‐Cy5.5 remaining within both the infiltrated IC21‐NR macrophages and the host tumor was calculated. Impressively, 2% of the tumor cells were CIB1‐siRNA‐Cy5.5+ (Figure 7b). Of the tumor infiltrated IC21‐NR macrophages 4% retained CIB1‐siRNA‐Cy5.5+, which is slightly lower than the 24 h time point in in vitro tumorsphere models (Figure 3d).

## 3 Conclusions

In this paper, we demonstrated that macrophages can horizontally transfer siRNA to tumor cells. We developed two reporter‐gene based models to characterize the pharmacodynamic siRNA transfer between macrophage and cancer cell populations. In addition, we confirmed macrophages horizontal transfer of siRNA can result in therapeutic effect as evidenced by decreased tumorsphere growth via delivery of CIB1‐siRNA to MDA‐MB‐468 breast cancer cells. Altogether, our data suggests that macrophages can deliver siRNA and that the delivery can be titrated through the initial amount loaded into macrophages and the ratio of macrophages. These models can be used in future studies to optimize the therapeutic effect of macrophage delivery systems.

There are several reasons that make macrophage horizontal gene transfer very worthy for exploring as a strategy in cancer gene therapy. First, the high ratio of macrophages might be achievable in the tumor environment where macrophages reportedly reside in high ratios.^45^ Second, while this technique may not be beneficial for delivering a therapeutic that required near universal transfer for effect (i.e., p53, KRAS), it would be useful for delivering therapeutics that can alter the tumor microenvironment (i.e., matrix metalloproteinases, TBF‐beta). Third, the number of macrophages needed can be reduced via optimization of the macrophage transfection conditions. If it is possible to load more siRNA into the macrophage and control its release, then macrophage horizontal gene transfer becomes a very valuable delivery mechanism. Finally, the mere finding of gene transfers presents a revolutionarily new strategy for gene delivery into solid tumors, an environment that is difficult to infiltrate. It could also be a unique future strategy for drug delivery to metastatic sites, as inflamed monocytes and macrophages form premetastatic niche in the metastatic process.^46^

Our study found that M2 macrophages were better at transferring siRNA to cancer cells, due to the role macrophage activation plays a significant role in siRNA uptake and release. Macrophages polarized into an M1, proinflammatory phenotype exhibited higher uptake of siRNA lipoplexes and high lysosomal activity, which directly correlates with the known behavior of these macrophages. In contrast, M2, anti‐inflammatory activated macrophages exhibited lower uptake rates of siRNA lipoplexes and lower lysosomal activity. M2 macrophages produced more exosomes than M1 macrophages. Taken together, this evidence suggests that M2 activated macrophage are more efficient at horizontal gene transfer because of lower lysosomal degradation and higher exosomal secretion activity. The activation dependent role for gene transfer has large implications for gene delivery into the solid tumor. Macrophages and monocytes recruited to the tumors become alternatively polarized to immunosuppressive M2 type.^47^ Our findings suggest that this may become an additional trigger‐release mechanism for the horizontal gene transfer from the siRNA preloaded macrophages migrating to the tumors (“Trojan horses”) to the surrounding tumor cells. Several studies demonstrate that nanoparticle size, shape, and composition can modify macrophage activation.^48^ Future studies should explore how the design of nanoparticles can modulate macrophage activation in vivo.

Today, most clinical approaches to tumor gene delivery are limited to direct injections of the nuclei acid to the tumor.^49^, ^50^ Recently, extracellular vesicles (EVs) or exosomes have attracted considerable attention due their ability to target many cancers.^51^, ^52^, ^53^ There are several advantages to using macrophages as a delivery vehicle in comparison to exosomes. Exosomes can be loaded relatively well with proteins,^54^, ^55^, ^56^ but loading with nucleic acids is considerably more difficult due to aggregation and charge. Purification and isolation of exosomes from pretransfected cells is also challenging due to low yield and quality control issues.^53^, ^57^ But perhaps the best selling advantage is that use macrophages can allow for the site‐specific localization and sustained release of exosomes, which additionally may be triggered in tumor environment.

Future work should explore the potential of translating gene delivery using macrophages in a clinical setting. Macrophages reside in the tissue, however, monocytes, their bloodborne precursors are an ideal cell type for cell therapy because they are easily harvested from the bloodstream. Monocytes/macrophages present a competitive advantage for delivery into the tumor environment because of the high recruitment and proliferation that occurs during tumorigenesis.^2^, ^46^, ^47^ Furthermore, monocyte based cell therapies already exist in other disease contexts and have shown some efficacy.^58^, ^59^ Immunogenicity must be considered; however it is likely to be similar to other types of autologous cell therapies.^60^, ^61^, ^62^

The implications of macrophage horizontal gene transfer are valuable for the macrophage biology community and the gene delivery field alike. An appreciation for the contribution of macrophages to disease progression and regeneration processes has grown over the last decade. Several studies have shown that macrophages can act as drug release “depots.” In as early as 2000, liver macrophages (Kupfer cells) were reported to act as drug reservoirs for docetaxel nanoparticles.^63^ More recent studies have shown macrophage slow release with drug from nanoparticle engulfment.^64^, ^65^ Our study corroborates previous reports that macrophages can release engulfed materials but also adds to this literature by demonstrating that macrophages can also release genes that were artificially loaded. The data presented focuses on siRNA, however, it is likely that other nucleic acids (i.e., pDNA, mRNA, microRNA) can also be delivered in this fashion.^29^ Moreover, the finding that macrophage polarization modulates this release of siRNA inspires the design of nanoparticle carriers that can take advantage of this mechanism. This establishes a framework for macrophages as a cellular theranostic: macrophage activity can be used to study pharmacokinetics and pharmacodynamics of drug–tumor interactions as well as promote the specificity of gene delivery.

## 4 Experimental Section

*Cell Culture*: IC21 macrophages were maintained in Roswell Park Memorial Institute (RPMI) medium supplemented with 10% fetal bovine serum (FBS). RAW 264.7 macrophages were maintained in Dulbecco's modified Eagle's medium (DMEM) media supplemented with 10% FBS. Human THP‐1 monocytes were cultured in (RPMI 10% FBS. THP‐1 monocytes were incubated with 20 ng mL^−1^ phorbol 12‐myristate 13‐acetate (PMA; Sigma) in RPMI 10% FBS media overnight to different into macrophages.

MDA‐MB‐231 breast cancer cells were lentivirally transduced to express green fluorescent protein (MDA‐MB‐231‐GFP). 4T1 breast cancer cells were lentivirally transduced to express renilla luciferase (4T1‐RLuc). 3LL lung cancer cells were lentivirally transduced to express firefly luciferase (3LL‐FLuc). All cancer cells lines (MDA‐MB‐231‐GFP/Fluc, 4T1‐RLuc, MDA‐MB‐468, 3LL‐FLuc) were maintained in DMEM media supplemented with 10% FBS. All cells were cultured under standard cell culture conditions (37° C, 5% CO_2_).

*CCK‐8 Assay*: CCK‐8 assay (Dojindo) was used to measure viability. Forty‐eight hours after transfection, media was removed from each well and replaced with a solution containing 90 µL of DMEM and 10 µL of CCK8 solution. CCK8 solution was allowed to incubate for 4 h to allow time for reaction to occur. Absorbance was measured at 450 nm using Spectromax M5 (Molecular Devices).

*siRNA Sequences: S*ilencer Firefly Luciferase (GL2 + GL3) siRNA is directly purchased from ThermoFisher (AM4629). Silencer Negative Control siRNA (referred to as control siRNA) is additionally purchased from ThermoFisher and used as control in the luciferase and time‐lapse imaging experiments. All other siRNA sequences were custom synthesized by Dharmacon Inc. (GE Dharmacon). The nonhomologous scrambled siRNA (referred to as siRNA‐Cy5.5) sense sequence is 5′ A.A.U.U.C.U.C.C.G.A.A.C.G.U.G.U.C.A.C.G.U.Cy5^5‐3′ 3′. The CIB1‐siRNA sense sequence is 5′ C.A.G.C.C.U.U.A.G.C.U.U.U.G.A.G.G.A.C.U.U.U.U.Cy5^5‐3′ 3′.

*Lipoplex Transfection*: C57Bl6 IC21 macrophages or Balb/c RAW 264.7 macrophages were transfected with siRNA using geneSilencer (Genlantis) transfection reagent in serum free media following the manufacturer's protocol. One million cells were seeded in 6 well in RPMI media supplemented with 10% FBS (without antibiotic) overnight. The following day, the media was replaced with 1 mL of RPMI serum free media (SFM). From 0.200 to 4 µg of siRNA was mixed with geneSilencer reagents and incubated for 15 min then added to 1 mL RPMI SFM. After 4 h of incubation, the transfection media was removed from cells and then macrophages were washed once in phosphate‐buffered saline (PBS) and twice in 1 mg mL^−1^ heparin sulfate in PBS to remove extracellular nanocomplexes. Cells were then removed from the tissue culture surface using a disposable tissue scraper. Cells were centrifuged at 800 rpm for 5 min to remove cell debris and resuspended in media in preparation for cell counting and coculture. Macrophages were either harvested 24 h after transfection or immediately after transfection.

*Electroporation siRNA Transfection*: Macrophages were electroporated adhering sample preparation protocols of the Neon Transfection System (ThermoFisher Scientific). 10e^6^ macrophages were resuspended with 2 µg siRNA and 100 µL resuspension buffer. Macrophages were electroplated using 1700 pulse voltages and 20 pulse width. Immediately following electroporation macrophages were placed in warm, prepared plates for downstream experiments.

*Tumorsphere Formation: F*or coculture tumorspheres, 5000 macrophages and 5000 cancer cells were transferred to 96 well ultralow attachment plates (Corning) and then aggregated by centrifugation at 1500 rpm for 15 min. Tumorspheres were cultured in 200 µL of DMEM supplemented with 10% FBS at 37 °C and 5% CO_2_ and 50 µL of fresh media was added to the side of each well every 3 days.

*Negative Stain TEM*: siRNA lipoplex samples were applied onto negatively glow‐discharged carbon‐coated grids (400 mesh, copper grid) for 1 min, and excess liquid was removed by blotting with filter paper. Freshly prepared 1.5% uranyl formate (pH 5) was added (5 µL) for 1 min and then blotted. Digital micrographs were collected using JEOL JEM 1230 Transmission Electron Microscope operated between 40 and 120 kV and equipped with 5‐axis goniometer stage in *X*, *Y*, *Z*, +/− 45° tilt, 360° rotation Gatan Orius SC1000 CCD camera. The images were recorded using Gatan Microscopy Suite 3.0 software.

*Flow Cytometry*: The percentage of macrophages expressing siRNA‐Cy5.5 was measured using flow cytometry (LSR II Fortessa; BD Biosciences). Cells transfected with unlabeled control siRNA were measured and used as a baseline for background fluorescence. Fifty thousand cells were measured per sample. Analysis of flow cytometry data was performed using FCS Express.

*ImagestreamX Imaging Flow Cytometry*: Flow cytometry analytics and fluorescent images of cells were collected using ImagestreamX (Millipore). Images were collected at 60× magnification. Ten thousand cells minimum were analyzed for each sample. Analysis of cellular fluorescence was performed using IDEAS (Millipore) software. Specifically, colocalization analysis was done using the bright similarity detail algorithm and the number of compartments in individual cells was analyzed using the Spot Count algorithm.

*Ultracentrifugation Exosome Isolation*: Macrophages grown in T175 flasks were incubated with serum‐depleted media for 24 h. The exosomal media is collected centrifuged at 1500 × *g* for 10 min to remove cells and large cell debris. The pellet was discarded, and the supernatant was centrifuged at 20 000 × *g* to remove larger debris and intact organelles. The resulting supernatant is then centrifuged at 150 000 × *g* to pellet exosomes.^53^ The recovery of exosomes was quantified by NTA.

*PEG Precipitation Exosome Isolation*: Macrophages grown in T175 flasks were incubated in serum free media for 24 h. Media was collected, and exosomes were isolated using gradient centrifugation. In brief, the culture supernatants were cleared of cell debris and large vesicles by sequential centrifugation at 1500 × *g* for 10 min and 4600 × *g* for 30 min. Supernatant was further filtered using through 0.2 µm syringe filters and mixed with 10% PEG overnight. Next, the media/PEG mixture was centrifuged at 4600 × *g* for 1 h to pellet the exosomes. The media was removed, the pellet was resuspended in 100 µL 1× PBS buffer, and stored at −20 °C until analysis. The recovery of exosomes was quantified by NTA.

*Breast Tumor Implantation and FACS Tumor Analysis*: MDA‐MB‐468 (5 × 10^6^ cells) in PBS were mixed 1:1 with matrigel (Corning) and injected into the left flank mammary fat pad of 5‐week‐old female nude mice (Jackson Laboratories, Bar Harbor, ME). Mice were injected with CIB1‐siRNA transfected IC21 macrophages after 5 weeks of tumor growth. Tumors were resected 24 h post macrophage injection for further analysis for presence of CIB1‐siRNA in the tumor. Tumors were minced and digested in 0.2% collagenase IV in DMEM at 37 C for 45 min. The resulting mixtures was strained through a 70 µm filter then incubated with ACK lysis buffer to eliminate remaining red blood cells. Cells were centrifuged to remove remaining cell debris and resuspended in FACS staining buffer before flow cytometry analysis. All animal procedures were approved by the University of North Carolina at Chapel Hill Institutional Animal Care and Use Committee (IACUC).

*Bioluminescence Assays*: After transfection, transfected macrophages were cocultured in 96 well plates with either 4T1‐RLuc, MDA‐231‐Fluc, or 3LL‐FLuc cancer cell lines. At various time points, the media was removed and 30 µL of cell lysis buffer (Promega) was added to the plate. The plates were wrapped in parafilm and stored in the freezer until measurement. Ten microlieter sample lysis mixture was analyzed for bioluminescent activity (Glomax 20/20 Luminometer) using either QunatiLuc (Invivogen) for renilla luciferase or Luciferin (Promega) for firefly luciferase. Luminescence measurements were normalized using measurements of total protein concentration using the BCA Protein assay (Thermo Fisher Scientific).

*Macrophage Activation*: Balb/c RAW 264.7 and C57Bl6 IC21 macrophages were plated at a density of 1.0 × 10^6^ mL^−1^ in a 6 well plate in 2 mL of activation media for 24 h. The activation conditions were: M0 (naïve, RPMI +10% FBS media only), M1 (1 µg mL^−1^ LPS and 20 ng mL^−1^ IFN‐γ in RPMI supplemented with 10% FBS), and M2 (20 ng mL^−1^ IL‐4 and 20 ng mL^−1^ IL‐13 in RPMI supplemented with 10% FBS). CCM is media collected from MDA‐MB‐468 human breast cancer cells. After activation, media was changed and replaced with serum‐free RPMI in preparation for experimentation. Macrophage polarization was validated by measuring mRNA expression of genes commonly upregulated during activation. M2 activation levels were assessed using CD206 mannose receptor and Arg1 while M1 activation levels were assessed using iNOS, TNFα, and CD86 (Figure S8, Supporting Information). In all other experiments if not specified differently the naïve unconditioned macrophages were used.

*Real‐Time PCR*: mRNA gene expression was determined using SYBR green quantitative real‐time PCR (qt‐PCR) on cDNA template. cDNA was generated from 1000 ng RNA per sample using oligo(dT)_12‐18_ primers and iScript cDNA Synthesis Kit (Biorad), according to manufacturer's instructions. Product was amplified with 20 × 10^−6^ m forward and reverse primers of gene of interest and SybrGreen Mastermix (Life Technologies) on an Applied Biosystems 7900 real‐time PCR. The primer sequences for SybrGreen primer sets were listed in the Supporting Information (Table 1).

*Live Cell Imaging*: Tumorsphere time‐lapse images were collected using the Incucyte (Essen Bioscience), a microscope and incubation system that allows for long‐term monitoring of live cells. Brightfield images were taken at 10× objective every 2 h. For the tumorsphere inhibition studies the growth of the tumorspheres was monitored using IncuCyte (Essen Bioscience), an incubator and built‐in microscope live‐cell imaging system. IncuCyte software was used to calculate the confluence, or percentage of the image covered by the tumorsphere. The confluence was used to calculate the percentage growth change over time using the following equation

(1) $$\text{Percentage}\;\text{growth}\;\text{change}\;=\frac{\text{Confluence}\left(t\right)-\text{Confluence}\left(t=0\right)}{\text{Confluence}\left(t=0\right)}\;\times \;100$$

## Conflict of Interest

The authors declare no conflict of interest.

## Supporting information

Supplementary
