# Supplementary material for: Targeted Delivery of siRNA Lipoplexes to Cancer Cells Using Macrophage Transient Horizontal Gene Transfer
Source: Adv Sci (Weinh). 2019 Sep 4;6(21):1900582. doi: 10.1002/advs.201900582 (PMC6839649; doi:10.1002/advs.201900582)
Supplement: Supplementary file 1 — Supplementary [file ADVS-6-1900582-s001.pdf]

Copyright WILEY-VCH Verlag GmbH & Co. KGaA, 69469 Weinheim, Germany, 2019.

ADVANCED SCIENCE
Open Access

Supporting Information

for *Adv. Sci.,* DOI: 10.1002/advs.201900582

Targeted Delivery of siRNA Lipoplexes to Cancer Cells Using Macrophage Transient Horizontal Gene Transfer

Elizabeth C. Wayne, Christian Long, Matthew J. Haney, Elena V. Batrakova, Tina M. Leisner, Leslie V. Parise, and Alexander V. Kabanov**

1. Supporting Information

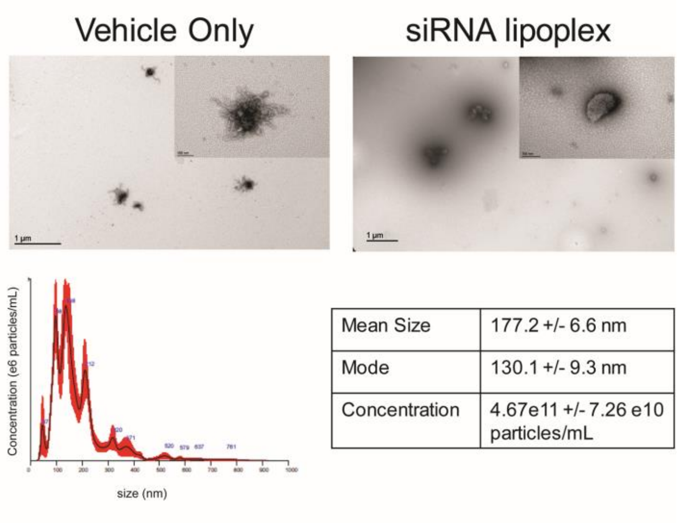

Figure S1 Negative TEM Stain analysis of siRNA lipoplex. Image of Vehicle (left) lipid formation which condenses into a lipoplex (right) upon introduction of siRNA. Nanoparticle Tracking Analysis (NTA) measurement of size distribution (177.2 +/- 6.6nm) and concentration (4.67e11 +/- 7.26e10 particles/mL) of siRNA lipoplexes.

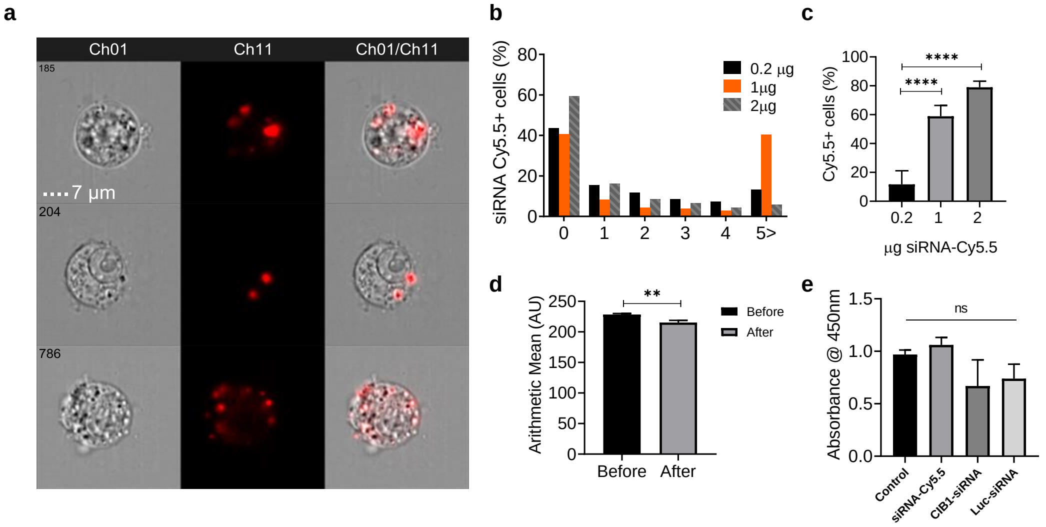

Figure S2 Characterization of siRNA loading into RAW 264.7 macrophages. a) Imaging Flow Cytometry (Imagestream) image of IC21 macrophages loaded with scrambled siRNA-Cy5.5. Objective 60x; scale bar 7 µm. b) Histogram of the percent of cells with different numbers of compartments (vesicles) containing scrambled siRNA-Cy5.5 24 hours after transfection. c) 1 x10^6 IC21 macrophages were plated in a 6-well plate transfected with varying concentrations of scrambled siRNA-Cy5.5+ 0.2µg, 1µg, 2µg. FACS analysis of siRNA-Cy5.5 remaining in macrophages 24hrs after loading. d) FACS analysis of mean intensity of macrophages transfected with scrambled siRNA-Cy5.5 before and after wash with 1mg/mL heparin sulfate. e) CCK-8 Cytotoxicity assay 10 x10^4 IC21 macrophages were plated into a 96-well plate and loaded with either scrambled siRNA-Cy5.5, Luc siRNA, or CIB1-siRNA. Statistical analysis done using Prism software by (c, e) one-way ANOVA followed by post-hoc Dunnett test. (d) Un-paired t-test for two group comparisons. **p<0.001, ****p<0.0001; n = 3 for all groups.

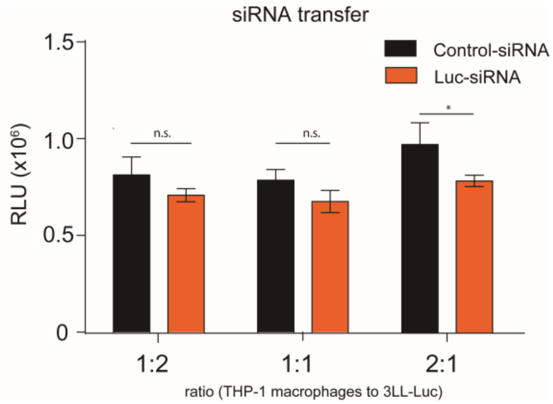

Figure S3 THP-1 macrophages siRNA transfer. 1.0 x10^6 THP-1 monocytes were treated with PMA for 24hrs to differentiated into macrophages. Following differentiation, THP-1 macrophages were transfected with 2µg of Luc siRNA (Thermofisher), washed and co-cultured with 3LL-Luc cells at varying ratios (2 monocytes: 1 cancer cells). Bioluminescence activity measured after 48hrs of co-culture. Statistical analysis done using Prism software. Unpaired t-tests. n=3 for each sample group. *p<0.05; n.s. = non-signifcant.

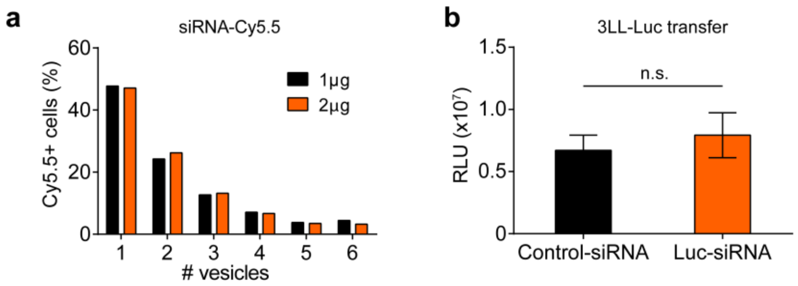

Figure S4 THP-1 monocytes siRNA transfer. a) 1.0 x10^6 THP-1 monocytes were transfected with either 1µg or 2µg of scrambled siRNA-Cy5.5 using geneSilencer (Genlantis) cationic lipoplexes. 24 hours after loading, THP-1 were analyzed using ImageStream to analyze the distribution of siRNA within cells. b) THP-1 monocytes were transfected with 2µg of Luc siRNA (Thermofisher) monocytes were co-cultured with 3LL-Luc cells at a 2:1 ratio (2 monocytes: 1 cancer cells). Bioluminescence activity measured after 48hrs of co-culture. Statistical analysis done using Prism software. Unpaired t-tests. n=4 for each sample group n.s. = non-significant.

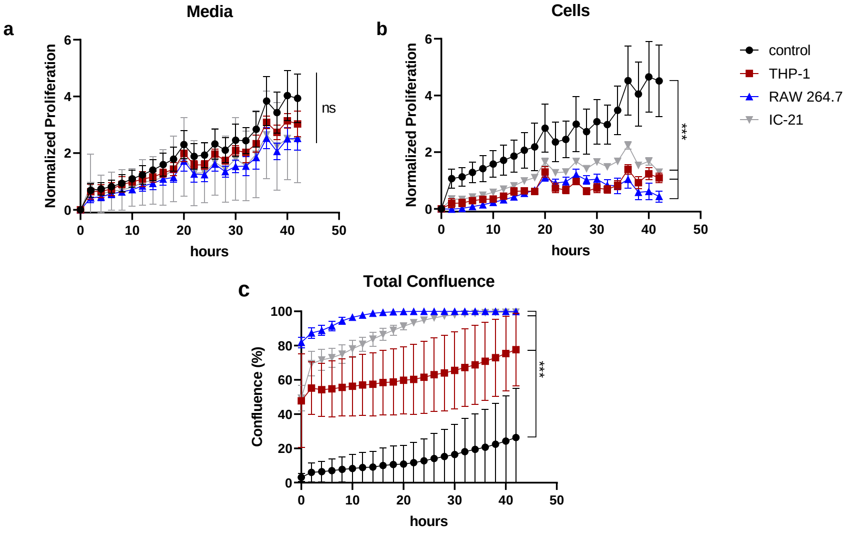

Figure S5 Effect of mouse macrophage co-culture on the viability of human MDA-MB-231 breast cancer cells. 10,000 MDA-MB-231 GFP human cancer cells were plated into 96-well plates. Normalized Proliferation represents time-lapse measurement of GFP object confluence normalized to the amount of GFP present in the initial timepoint. a) Cancer cells were either cultured with DMEM, with conditioned media from human macrophage (THP-1, red), mouse Balb/c macrophages (RAW 264.7, blue) or C57Bl6 mouse macrophages (IC-21, gray). b) Cancer cells were either cultured alone (control, black) or with human macrophage (THP-1, red), mouse Balb/c macrophages (RAW 264.7, blue) or C57Bl6 mouse macrophages (IC-21, gray). 20,000 macrophages were added to cancer cells to make the ratio of macrophages to cancer cells 2:1. c) Time-lapse data of confluence measured by phase contrast. Statistical analysis done using Prism software by *(a-c)* one-way ANOVA followed by post-hoc *Dunnett* test. ns= non-significant, ***$p<0.001$; n=3 images per well, n = 3 wells for each groups.

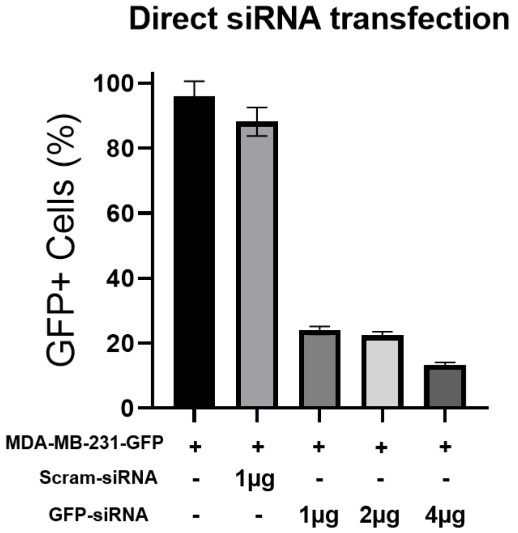

Figure S6 Direct GFP-siRNA transfection of human MDA-MB-231 breast cancer cells. 7.0×10^5 MDA-MB-231 GFP human cancer cells were plated into 6-well plates. Cells were transfected with GFP-siRNA using geneSilencer lipoplexes according to the manufacturer protocols. 48 hrs after transfection, cells were harvested and GFP intensity was measured using flow cytometry. The percentage of GFP+ cells were gates based on the non-transfected MDA-MB-231-GFP cells intensity profile.

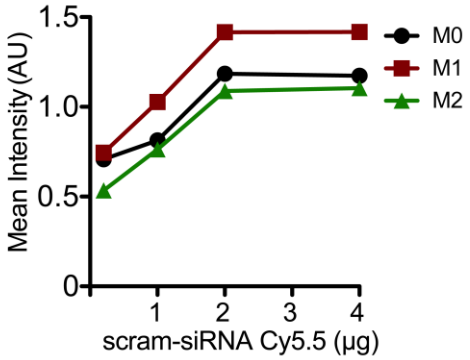

Figure S7 Uptake of siRNA in activated macrophages as measured by fluorescence mean intensity Naïve, M1 (1ug LPS) and M2 (20ng/mL IL-4) conditioned IC21 macrophages were transfected with varying amounts of scrambled siRNA labeled with Cy5.5 (scram-siRNA Cy5.5). Mean intensity of Cy5.5 fluorescence measured immediately after transfection.

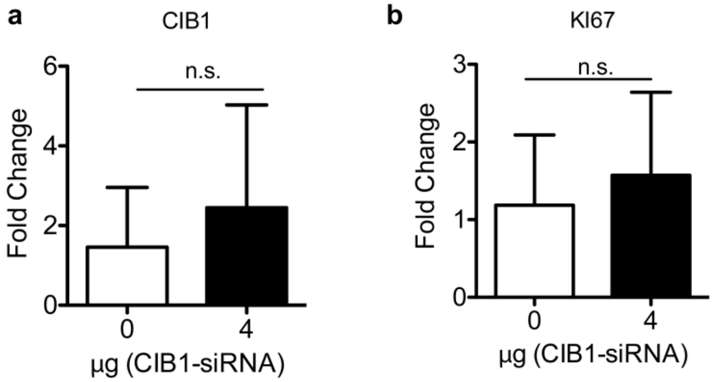

Figure S8 IC21 macrophages loaded with CIB1-siRNA does not affect CIB1 expression or proliferation in MCF10a cells. IC21 macrophages were loaded with 4µg of CIB1-siRNA and co-cultured in sphere formation with MCF10a (normal human epithelial breast cells). After 4 days, spheroids were lysed and assessed for changes in mRNA expression of CIB1 and KI67. Statistical analysis done using Prism software. n=3 for each sample group. Unpaired t-tests. n.s. = non-significant.

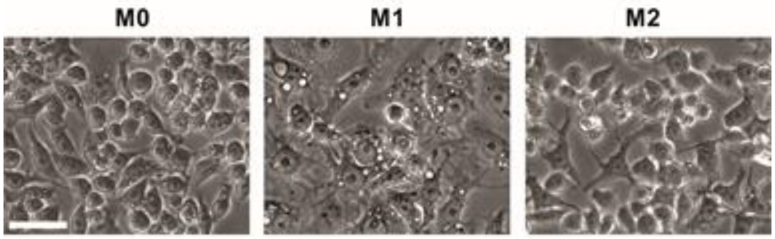

	M0	M1	M2
ARG1	1.0	47.671	73148.2
CD163	1.0	ND	14.3
NOS2	1.00	20.786	14.2
TNFA	1.0	34.220	24.0

Figure S9 qPCR Analysis of IC21 macrophage activation profile. Chart of mRNA expression of several genes known to be upregulated in macrophage activation. M1 profiles are highlighted in red and M2 is highlighted in green. Fold change was compared to naïve M0 macrophages and calculated using the comparative CT method.
